# Occupational health literacy and work ability: a moderation analysis including interpersonal and organizational factors in healthy organizations

**DOI:** 10.3389/fpubh.2024.1243138

**Published:** 2024-02-07

**Authors:** Julian Friedrich, Maylin Rupp, You-Shan Feng, Gorden Sudeck

**Affiliations:** ^1^Institute of Sports Science, University of Tübingen, Tübingen, Germany; ^2^Institute of Occupational Medicine, Charité—Universitätsmedizin Berlin, corporate member of Freie Universität, Berlin and Humboldt-Universität zu Berlin, Berlin, Germany; ^3^Institute for Clinical Epidemiology and Applied Biometry, University Hospital and Faculty of Medicine, University of Tübingen, Tübingen, Germany

**Keywords:** health, work and occupation, health promotion, occupational health literacy, health-oriented leadership, participation, values of health, latent regression analysis

## Abstract

**Introduction:**

Healthy organizations approach to occupational safety and health should holistically include individual, interpersonal, and organizational levels. There is an empirical research gap in considering different levels in organizations for health promotion in the context of maximizing work ability. This study aims to investigate the association of (1) occupational health literacy (on an individual level), (2) health-oriented leadership (interpersonal level), (3) participation possibilities in health, and (4) values of health in companies (both organizational levels) on work ability. Additionally, we examined the potentially moderating role of health-oriented leadership, participation possibilities in health, and values of health between occupational health literacy and work ability.

**Methods:**

Cross-sectional data were obtained from 828 employers and employees in small and medium-sized enterprises. Self-report measures included occupational health literacy, health-oriented leadership, work ability, participation possibilities in health at work, and values of health in the company. Occupational health literacy comprises two factors: a knowledge-/skill-based approach to occupational health and a willingness/responsibility for occupational health. Participation possibilities in health are measured regarding participatory opportunities and co-creation of health at work. Values of health in the company capture the importance of health in the workplace and the scope for improving employees’ health. Data were analyzed using latent regression and latent moderation analyses controlling for age, gender, and educational level.

**Results:**

Occupational health literacy (knowledge-/skill-based), health-oriented leadership, participation possibilities in health, and values of health in companies showed positive associations with work ability. Health-oriented leadership on an interpersonal level was found to moderate the positive relationship between (knowledge-/skill-based) occupational health literacy and work ability. Participation possibilities in health on an organizational level acted as a moderator on the relationship between both occupational health literacy factors and work ability.

**Discussion:**

Individual, interpersonal, and organizational factors play important roles in maintaining work ability in healthy organizations. This study highlights the importance of promoting occupational health literacy among employees and leaders, creating a healthy workplace through health-oriented leadership, and providing participatory opportunities for co-creation in health promotion at work. Future research should further explore these factors’ roles in different industries and contexts and how they may be addressed effectively in tailored workplace interventions.

## 1 Introduction

The modern workplace faces complex challenges due to increasing digitalization and changes in social and ecological conditions (1, 2). Considering the modernization in work environments and the socio-demographic development, there should be a stronger focus on – and more company responsibility for—maintaining employee health and sustainable work ability (3, 4).

A healthy organization (5, 6) requires ensuring an environment promoting employee health and organizational effectiveness (7, 8). Work can design resources for individual employees but can also contribute to work-related illnesses and increase the risk of chronic diseases and mental disorders (9). For a holistic approach to health, it is important to address the individual, the interpersonal, and the organizational level in companies to consider health comprehensively (6).

A conceptual model includes integrated approaches to the protection and promotion of employee health and safety (10). Knowledge and skills about occupational health form important worker proximal outcomes. On the one hand, nurturing individual progress leads to skill development and increased employee competencies. On the other hand, psychosocial factors or the work organization create conditions for health at work that enable a person to develop competencies and engage in a healthy way in the organization (10). Both, individual and organizational factors can promote health and work ability in the long term (11, 12).

### 1.1 Individual outcome: work ability

Work ability is the functional ability of employees to meet the job’s requirements with respect to health and resources and is an often included concept in validated measurement instruments (13). It captures the dynamic between job demands and an individual’s health or competencies (14). In a person-oriented, longitudinal approach, the trajectories of work ability were studied: Younger age, upper management position as well as job control or organizational climate predicted a positive development of work ability (15).

Furthermore, in the conceptual model of Sorensen et al. (9), the conditions of work and worker proximal outcomes directly influence other worker outcomes. Lower perceived work ability was, e.g., associated with higher long-term sickness absence and a higher risk of early retirement (16, 17). These negative individual worker outcomes produce higher healthcare costs and can affect the organization in the long term (10). Therefore, the psychosocial work conditions in organizations should be more considered (15). Nevertheless, the prevention of a decline in work ability is an important concept in healthy organizations and an essential individual employee outcome (18). When promoting work ability, on the one hand, organizational conditions should be addressed, so that the employees experience less occupational demands and hazards to work in a healthy way (19). On the other hand, individual skills should be improved to address the occupational competence of employees so that they can manage their work tasks (20).

### 1.2 Individual level factor: occupational health literacy

Health literacy includes the knowledge and skills to meet complex health demands in different living environments (21). Competencies are context- or situation-dependent (22) and health literacy is functionally applied in specific environments (23). Occupational health literacy (OHL) more narrowly focuses on people’s knowledge, skills, and readiness to access and process health-related information and apply it in work situations (24). OHL includes two dimensions, (1) a knowledge- and skill-based approach to health and (2) a willingness and responsibility for occupational health (25). High OHL can empower people to make health-related decisions in the work environment (26). Furthermore, OHL is modifiable (27) via interpersonal support as well as organizations that support healthy behaviors and consider employee health holistically (6).

Work ability has been shown to be positively correlated with higher general health literacy (28), with nearly 20 percent of its variance explained by health literacy (29). In addition, limited health literacy has also been shown to contribute to social inequality and inequity in health (30–32). These studies assessed the relationships with a general health literacy measure in worker samples and not specifically an OHL scale. Nevertheless, better health information processing as well as a proactive approach to health were related to work ability (29, 33, 34).

### 1.3 Interpersonal level factor: health-oriented leadership

From an occupational health perspective, leadership is not a neutral element but can be an important factor in work-related health outcomes in organizations (35). It is an indicator to ensure the health of employees in the workplace and the availability of resources (35). Leaders represent or shape the organizational structures and work characteristics (36) due to their influential roles (37), and their behavior is related to the health of those led (35, 38).

Although different leadership styles exist, a domain-specific leadership style such as health-oriented leadership (HoL) supports a positive and direct impact on follower health (35, 39). Leaders’ work demands and stresses are often considered a risk factor for employees (40). If leaders have fewer resources, they are less able to support those under their leadership and add stress to those they lead in the form of pressure. Destructive leadership practices such as abusive supervision or absences influences work ability negatively. In contrast, in other leadership styles, leaders are convincing through their role model function (41). Transformational leaders positively stimulate and inspire followers to achieve their goals. In transformational leadership, leaders empower the followers to develop and grow by responding to individual follower’s needs (41). In HoL, however, the inspiration for healthy action is in the foreground, which is in line with health behavior changes at the workplace (35). Therefore, we selected HoL for associations with work ability in this study.

Health-oriented leadership captures self-directed health-promoting leadership (SelfCare) and follower-directed health-promoting leadership (StaffCare). The concept provides an integrative approach for assessing leader and follower perspectives and differentiates between the dimensions value of health, health awareness, and health behavior. Existing research supports the positive effects of HoL in routine working conditions (42, 43). It was noted that strengthening the health literacy of leaders is essential for their own health behaviors and even so for employees’ health (44, 45). Data on combining HoL and health literacy as individual and interpersonal factors are scarce (46, 47). This research gap calls for health-promoting changes through OHL (48) as well as HoL (49) in line with the call for maintaining and promoting work ability (26).

### 1.4 Organizational level factors: participation possibilities and values of health in companies

It is increasingly important for organizations to facilitate employee participation in workplace health as a part of health promotion. For this, it is necessary to involve employees at the group and organizational level to achieve long-term improvement in working conditions (50). In this context, participation possibilities in health at work can be understood as a process that allows employees to exert influence over their work, the conditions of it (51), and also in their health at work within co-creating healthy working conditions for employees. Possibilities for employees to shape their work and stronger participation offers can represent health-promoting potentials that contribute to a healthy organization, especially in sectors with high job demands (43). The use of participatory approaches also aligns research linking working conditions, work environment practices, and employee participation (52, 53). The participation of employees in shaping their health-promoting work environment supports the implementation of measures tailored precisely to their needs. Personal responsibility for occupational health is increasingly crucial in shaping healthy work environments. For this reason, among others, individuals need OHL and participation opportunities to take advantage of a health-oriented work environment (54). In a study sampling younger workers, increased social support was positively related to work ability (55). This underlines the necessity of a good organizational climate and support following a health-oriented approach in companies, such that work ability can be improved in the long term (15, 56).

In addition, the value of health in a company can be an important organizational factor. The value of health in a company highlights the extent to which health is seen as an important human and business value (57). In healthy organizations, health is a strategic company interest and is experienced by employees through the organizational structures, policies, and practices that shape the overall health within the workplace (9). While there is an association of healthy people with a healthy organization (57), on the one hand, employees have an own responsibility to remain able to work in the long term (4). On the other hand, health conditions can be influenced by a proactive and preventive workplace health promotion and a common mission statement regarding health (57) to help employees stay healthy and able to work. It is therefore a necessity for companies to create financial and health-promoting framework conditions that enable people to engage in a health-oriented way.

Establishing a culture of health, safety, and well-being is linked with a competitive business advantage (58). In addition to direct costs of health care for ill employees (59), there are indirect costs of lower productivity and reduced engagement and commitment (58, 60). The importance of the value of health in companies is also an essential attractor for skilled employees in times of labor shortages.

Moreover, there are structural differences regarding the company size in implementing a mission statement and systematic occupational health management (61). Bigger companies might have more personnel capacity and a higher budget for systematic strategies. A discrepancy occurs between the stated importance of health and implemented health activities or programs, especially in small and medium-sized enterprises (62). Therefore, the organizational framework conditions for occupational health matter and should be improved by structural and personal resources (63). Although workplace policies are anchored in the conceptual model for integrated approaches to the protection and promotion of worker health and safety (10), the model could be expanded to include the importance and values of health, so that the motivation to create health-promoting conditions is emphasized (64).

### 1.5 Research gaps, aims, and hypotheses of the present study

**Figure 1:**
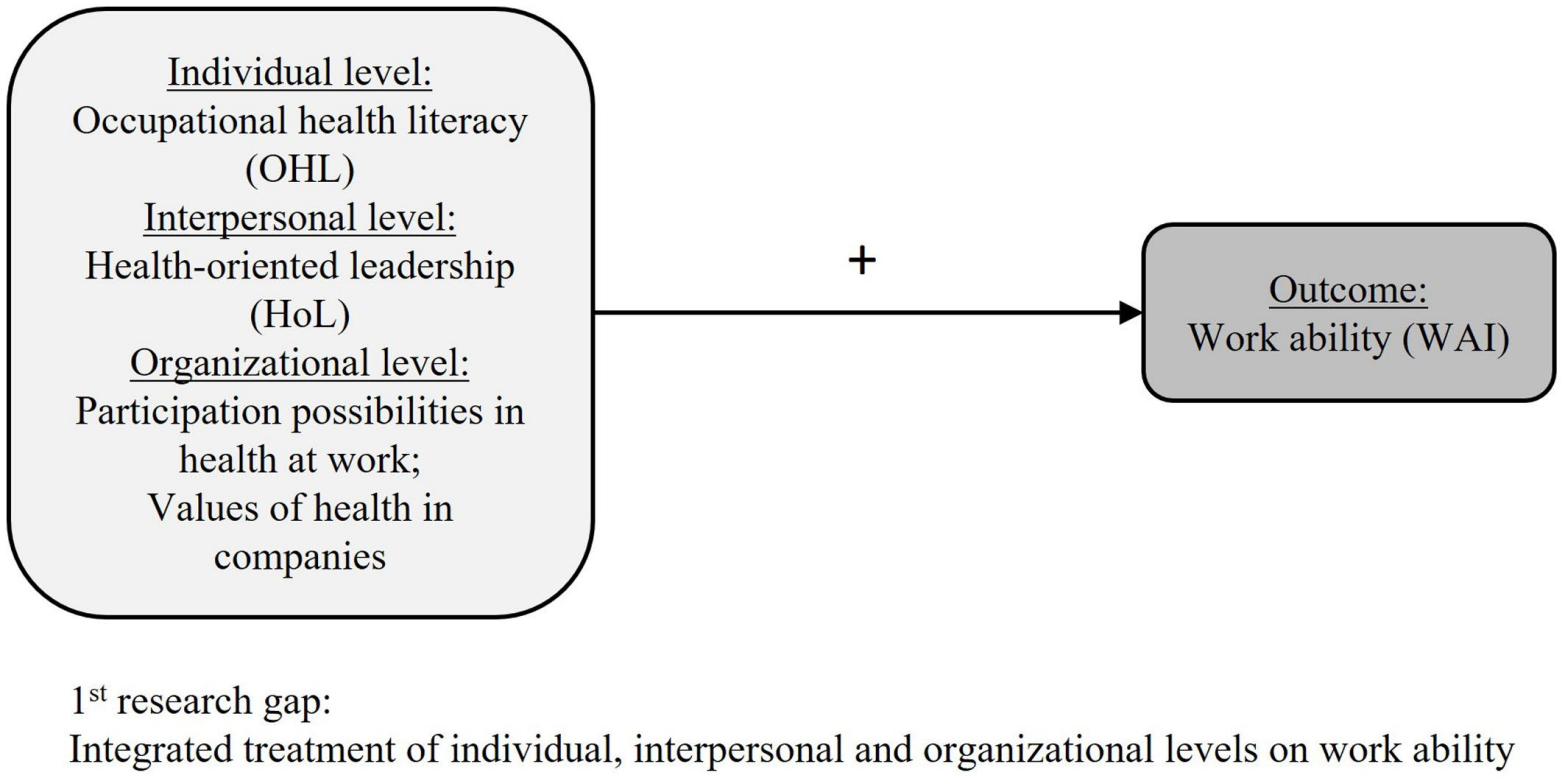
Research model for hypothesis 1.

Our theoretical assumption is based on the premise that integrated treatment of individual, interpersonal, and organizational levels will contribute to greater improvements in work ability than treating each pathway separately (9, 10). When considering the concept of healthy organizations, how individual, interpersonal, and organizational factors affect work ability is an empirical research gap. OHL and HoL are currently understudied in the empirical research of work ability (46, 47). To contribute to this research gap, first, it is important to draft individual competencies as well as interpersonal conditions such as HoL and examine the associations with workers’ outcomes (9), like work ability (13). Different factors are selected that can later be addressed consecutively. The chosen variables should, therefore, be related but not too similar and represent the different levels in an organization. One aim of this study is to examine the association of different factors on individual, interpersonal, and organizational levels with work ability in small and medium-sized enterprises in Germany as formulated in hypothesis 1 (see Figure 1):

H1: The individual (OHL) and interpersonal (HoL) factors, as well as the organizational factors (participation possibilities in health and values of health in companies), are positively associated with work ability when controlling for age, gender, education, and hierarchy level.

**Figure 2:**
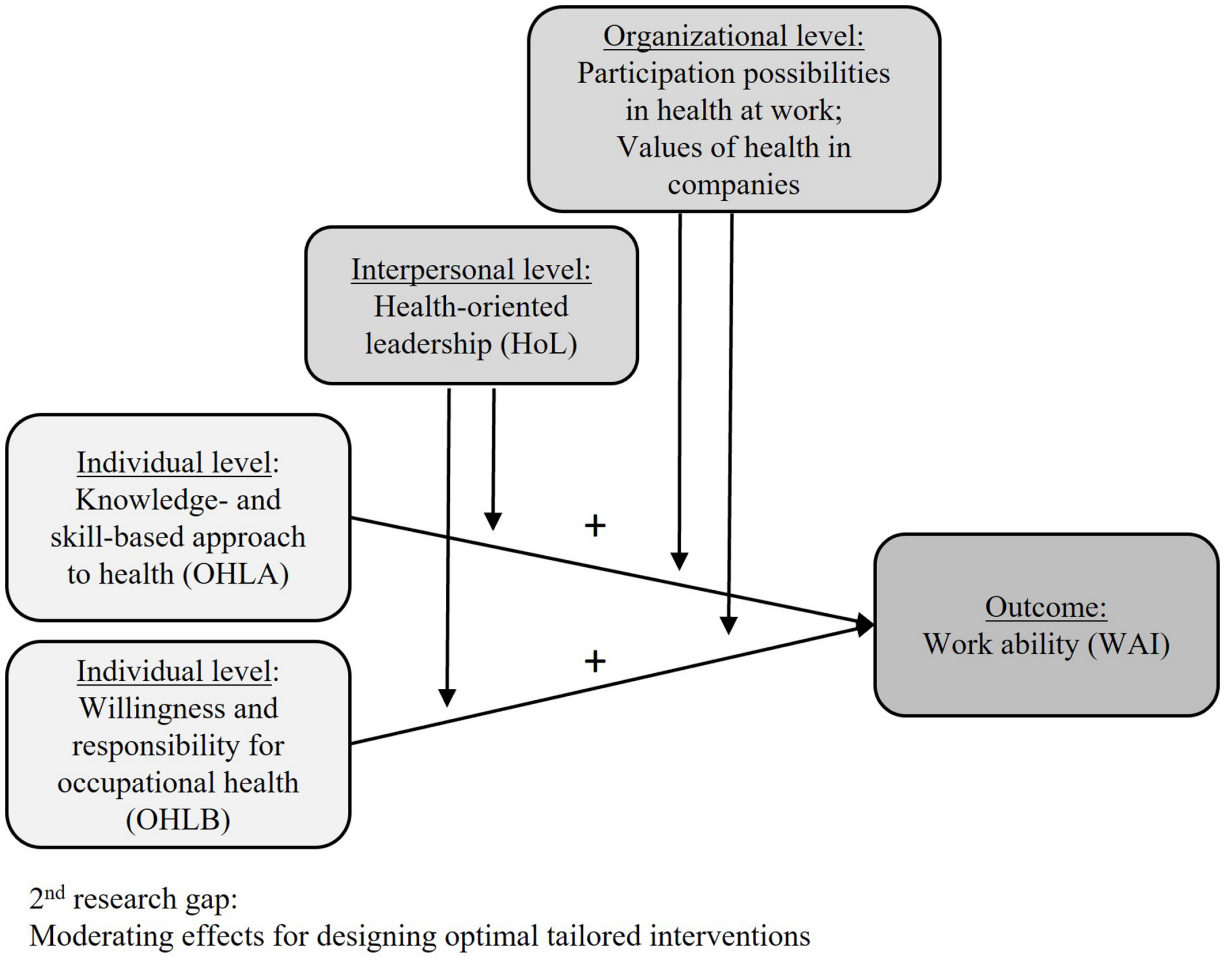
Research model for hypotheses 2 and 3.

Secondly, the interactions of the different variables should be examined and their joint effect on work ability should be investigated (see Figure 2). A deeper understanding of moderating effects on the different levels may help design optimally tailored interventions. In this context, another aim of this study is to explore the moderation effects of interpersonal and organizational factors on the relationship between OHL as an independent variable and work ability as an outcome at the individual level. HoL as interpersonal support can help to develop healthy organizations (7, 65). HoL is a crucial leadership approach to enable work-related autonomy and participation in the workplace (66). Participation possibilities and values of health in companies can change working conditions and in the long term can have positive impacts on work ability. Thus, the following hypotheses are:

H2: On an interpersonal level, HoL moderates the positive relationship between OHL and work ability.

H3: On an organizational level, participation possibilities in health and values of health in companies moderate the positive relationship between OHL and work ability.

## 2 Materials and methods

### 2.1 Data collection and sample

In a cross-sectional computer-assisted telephone survey (December 2020 to May 2021), *n* = 831 employers and employees in diverse small and medium-sized enterprises (< 249 employees) in Germany were interviewed. We excluded three participants due to language barriers and lack of concentration, resulting in *n* = 828 participants for data analyses. *A priori* sample size planning ensured sufficient power (significance level 5% two-sided, power 80%) to detect small effect sizes of 0.20. Educational level was stratified and approximately equally distributed. The hierarchy level was separated into employees without personnel responsibility and employees with personnel responsibility for at least one other person, employers, or supervisors. The industry types were based on the Federal Statistical Office in Germany. All demographic characteristics can be found in Table 1.

**Table 1:** Demographic characteristics of the study sample.

Characteristics	Participants *n* = 828
Age	
*M* (*SD*)	41.5 (12.2)
[*Min*, *Max*]	[18, 72]
Gender	
Female	53.7%
Male	45.0%
Diverse	1.2%
Educational level	
Lower secondary school leaving certificate or no certificate	31.1%
Secondary school leaving certificate	30.0%
Higher education entrance qualification	38.7%
Hierarchy	
Low: no personnel responsibility	72.0%
High: employee with personnel responsibility, employer or owner	28.0%
Sectors	
Service sectors	35.0%
Trade/hospitality/transport	23.9%
Manufacturing industry	22.2%
Administration and research	18.0%
Agriculture and forestry	0.7%

### 2.2 Measures

For the implementation of tailored occupational health interventions, it is crucial to have knowledge of different factors and their covariates which predict and influence work ability. The dependent variable of all analyses was *work ability*. We used the seven-item short version of the Work Ability Index (WAI) (67), for which responses can be combined into an overall score. Participants were asked about their current work ability in relation to demands, existing diseases and illnesses, estimated work impairment, work ability in the future, and mental capacities (67). Higher scores corresponded to a higher ability to work.

The primary independent variable was *occupational health literacy* (OHL), which was measured using the 12-item Occupational Health Literacy Scale (25). A two-factor structure was recommended (25), including a subscale with eight items for knowledge- and skill-based approach to health (OHLA) with response levels from 1 (very difficult) to 4 (very easy) and another subscale with four items for willingness to change and take responsibility for health (OHLB) with response levels from 1 (strongly disagree) to 4 (strongly agree).

*Health-oriented leadership* (HoL) was modeled as a moderator to test different interactions on an interpersonal level. Three items modified from the Health-oriented Leadership Scale (68) captured the perceived HoL from employees’ perspectives. Because HoL consists of two dimensions, SelfCare and StaffCare, *n* = 587 employees without personnel responsibility answered the items on perceived StaffCare of their supervisor. For economic reasons, only the dimensions of awareness and value of health were chosen, and all three items were slightly modified. The items were: (1) “My supervisor regularly checks to see if anything is wrong with the employees’ health” (awareness), (2) “My supervisor feels responsible for the health of the employees” (value), and (3) “My supervisor attaches great importance to health in the workplace” (value). Answers were given on a scale from 1 (strongly disagree) to 5 (strongly agree).

*Participation possibilities in health at work* were assessed with a self-constructed scale because no validated scale currently exists. We developed three items to measure this concept, which had an acceptable internal consistency (Cronbach’s α = 0.82). The scale measured participation opportunities with different items from 1 (strongly disagree) to 5 (strongly agree), i.e., (1) “I can have a say in matters related to my health at work,” (2) “In our company, there are many opportunities to participate in shaping a healthy work situation,” and (3) “Suggestions on health in the workplace are very welcome in our company.”

*Values of health in companies* were also measured with a self-constructed scale using two items on a scale from 1 (strongly disagree) to 5 (strongly agree). Higher scores indicated better-perceived conditions of occupational health in one’s own company. The first item captured changeable conditions at the workplace (i.e., “The conditions at my workplace make it possible to implement improvements with regard to health”). The second item was geared toward companies’ financial potentials for health promotion (i.e., “My company has the financial means to provide measures to promote employee health”). The average inter-item correlation was 0.36 in the ideal range between 0.15 and 0.50, so the items are correlated but do not measure the same construct (69). Cronbach’s α for this scale was 0.52. Because they are newly developed items, weighted scoring procedures are not available.

Regarding health, research shows differences and inequity due to socioeconomic factors or hierarchy levels. Therefore, socioeconomic and demographic factors should be considered to better control for social factors in statistical models.

In line with previous literature (70), we measured the *socioeconomic status* with professional qualification, professional status, and household net income. These responses were then weighted as suggested by Lampert et al. (63) and the socioeconomic status index could range between 1 and 7. Due to missing values on net income, for which statistical implications would arise from imputation (71), we used the educational dimension with school leaving certificates as the primary measure for socioeconomic status in these analyses. Educational status was measured with the highest attained education level, which in Germany is defined as (1) lower secondary school leaving certificate or no certificate, (2) secondary school leaving certificate, and (3) higher education entrance qualification. The educational status was coded and used as a continuous variable.

*Hierarchy levels*: We asked the participants whether they are employers or employees and, if they are employed, whether they have personnel responsibility for at least one other person in the company. Some questions that addressed HoL were administered based on hierarchy levels: those with personnel responsibility responded to questions from a leader perspective, and those without personnel responsibility responded to questions from a dependent perspective. Therefore, a subsample with *n* = 596 (72.0%) employees without personnel responsibility was available in analyses with HoL.

### 2.3 Statistical procedure and analyses

Latent regression was chosen as the primary statistical approach because this approach does not rely on external weights to derive scale values. The *R* statistics version 4.1.3 with the package “lavaan” (72) was used for all analyses. A simple confirmatory factor analysis was used to pre-analyze the measurement models of the included scales.

To test *hypothesis 1*, we estimated several latent regression models using the dependent variable WAI and the independent variables OHL (on an individual level), HoL (on an interpersonal level), participation possibilities, and values of health in companies (both on an organizational level). For the analysis including HoL, the models were based on a subsample of employees without personnel responsibility: employers and supervisors were not asked about their perceived HoL. Including these participants with systematical missing values would have led to biases. In a separate latent regression analysis for organizational factors with all participants, participation possibilities in health and values of health in companies acted as independent variables.

To test *hypotheses 2 and 3*, we estimated latent moderation models with double mean centering (73) using the factors of OHL as independent variables on the dependent variable WAI for the moderators’ HoL, participation possibilities, and values of health in companies.

All models included the control variables age, gender, and educational level. Furthermore, we did not impute or use full information maximum likelihood (74) due to systematically missing values across hierarchy levels. Separate regression models were used to understand HoL. Additionally, due to the difficulty of implementing and interpreting complex interaction, as well as indications of collinearity for some of the measures, we did not combine the above-described models into a comprehensive regression model. For acceptable model fits, root mean square error of approximation (RMSEA) with a cut-off value lower than 0.08, standardized root mean square residual (SRMR) with a cut-off value lower than 0.06 (75, 76), comparative fit index (CFI), and Tucker-Lewis index (TLI) with acceptable values close to 0.90 were used (77, 78).

## 3 Results

### 3.1 Measurement models

A preliminary confirmatory factor model with the scales WAI, OHLA, OHLB, HoL, participation possibilities in health, and values of health in companies found a satisfactory model fit for the included scales: χ^2^(175) = 425.0, *p* < 0.001, CFI = 0.93, TLI = 0.92, RMSEA = 0.05, SRMR = 0.05. Significant correlations between the included scales were observed (see Supplementary Table S1). Furthermore, high covariances between HoL and participation possibilities, as well as values of health in companies, were found. Comprehensively, the correlation of the two organizational factors, participation possibilities and values of health, was strong. Due to these strong correlations and the theoretical and statistical dependence between the factors, we assumed multicollinearity for these scales. With higher multicollinearity, the precision of the estimated coefficients in a regression analysis is reduced and model interpretation can become ambiguous (79). Therefore, we conducted two separate latent regression analyses for individual/interpersonal and organizational factors.

### 3.2 Latent regression analyses

Good fit indices—χ^2^(135) = 337.6, *p* < 0.001, CFI = 0.93, TLI = 0.91, RMSEA = 0.05, SRMR = 0.05—were found for the latent regression analysis for individual/interpersonal factors (see Table 2). The factors OHLA and HoL were significantly associated with WAI after controlling for age, gender, and educational level. The factor willingness and responsibility for occupational health showed no significant association.

**Table 2:** Standardized regression coefficients (Stand coef.) and results of latent regression analysis with individual/interpersonal factors on work ability for employees (*n* = 517).

Factors	Stand. coef.	*SE*	*z*	*p*
Age	−0.35	0.02	−8.86	0.01
Gender	−0.03	0.41	−0.68	0.50
Educational level	0.06	0.12	1.55	0.12
Knowledge- and skill-based approach to health (OHLA)	0.25	0.46	4.78	0.01
Willingness and responsibility for occupational health (OHLB)	0.02	0.65	0.28	0.78
Health-oriented leadership (HoL)	0.16	0.23	3.27	0.01

Significant associations with WAI were also found for participation possibilities and values of health in companies on an organizational level (see Table 3), with satisfactory model fits: χ^2^(19) = 107.6, *p* < 0.001, CFI = 0.94, TLI = 0.87, RMSEA = 0.08, SRMR = 0.03. Therefore, hypothesis 1 was partly confirmed.

**Table 3:** Standardized regression coefficients (Stand coef.) and results of latent regression analysis with organizational factors on work ability for all participants (*n* = 776).

Factors	Stand. coef.	*SE*	*z*	*p*
Age	−0.30	0.01	−8.76	0.01
Gender	−0.00	0.33	−0.12	0.90
Educational level	0.07	0.10	2.10	0.04
Hierarchy level	0.10	0.24	2.74	0.01
Participation possibilities in health	0.21	0.41	2.91	0.01
Values of health in companies	0.16	0.41	2.16	0.03

### 3.3 Latent moderation analyses

Latent moderation analyses were conducted separately to test for the OHL factors and the interactions between each interpersonal and organizational factor on WAI. The analyses included no moderations between OHL and other individual factors. The interaction between the factors OHLA and HoL on WAI was significant (see Table 4), indicating a moderation effect and partly confirming hypothesis 2. For the factor willingness and responsibility for occupational health (OHLB), no significant interaction with HoL was found. The factor was therefore excluded from further moderation analyses to better interpret the results. The model fit was acceptable for some indices: χ^2^(729) = 1773.2, *p* < 0.001, CFI = 0.92, TLI = 0.90, RMSEA = 0.05, SRMR = 0.10.

**Table 4:** Standardized regression coefficients (Stand coef.) and results of latent moderation analysis with personal factors on work ability for employees (*n* = 517).

Factors	Stand. coef.	*SE*	*z*	*p*
Age	−0.34	0.02	−8.99	0.01
Gender	−0.03	0.41	−0.68	0.49
Educational level	0.06	0.12	1.57	0.12
Knowledge- and skill-based approach to health (OHLA)	0.25	0.46	4.67	0.01
Willingness and responsibility for occupational health (OHLB)	0.04	0.64	0.79	0.43
Health-oriented leadership (HoL)	0.15	0.23	3.20	0.01
OHLA*HoL^a^	−0.14	0.30	−3.64	0.01

^a^OHLA*HoL, interaction between the factor knowledge- and skill-based approach to health and health-oriented leadership.

**Figure 3:**
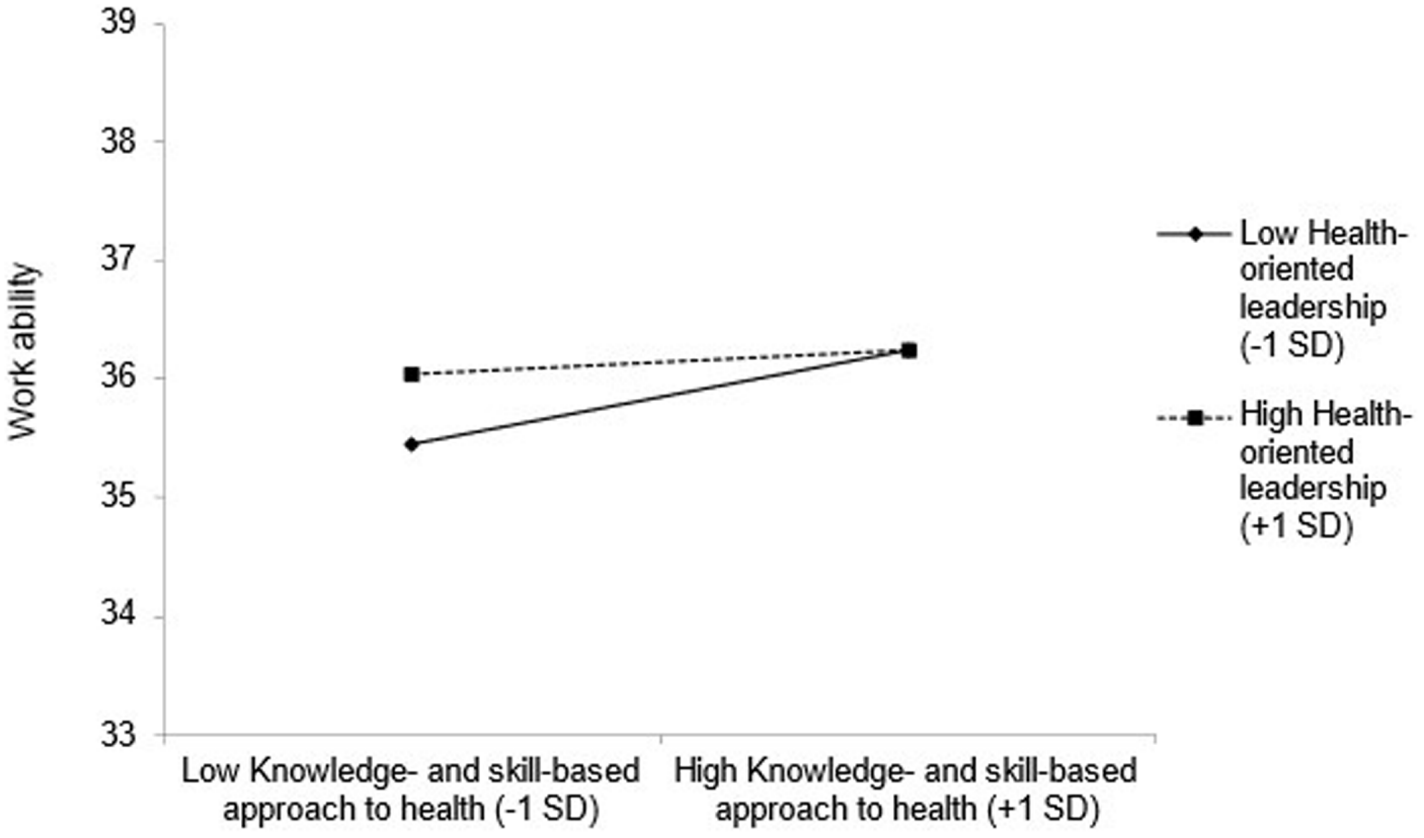
The moderating effect of the knowledge- and skill-based approach to health and health-oriented leadership on work ability.

The interaction was statistically significant among employees with lower HoL. The slope was steeper (the positive effect of OHLA was stronger) among employees with lower HoL (*B* = 3.29, *p* < 0.01) compared to those with higher HoL (*B* = 0.99, *p* = 0.08; see Figure 3).

Separate latent moderation models were estimated for OHL factors, participation possibilities, and values of health in companies. Fit indices for most moderation models met acceptable thresholds: χ^2^(1,307) = 3167.9, *p* < 0.001, CFI = 0.89, TLI = 0.87, RMSEA = 0.04, SRMR = 0.07. Participation possibilities had statistically significant moderating effects in the context of both OHL factors as independent variables and WAI as a dependent variable, partly confirming hypothesis 3 (see Table 5).

**Table 5:** Standardized regression coefficients (Stand coef.) and results of latent moderation analysis with participation in health on work ability for all participants (*n* = 722).

Factors	Stand. coef.	*SE*	*z*	*p*
Age	−0.29	0.01	−8.62	0.01
Gender	0.02	0.34	0.54	0.59
Educational level	0.07	0.11	2.02	0.04
Hierarchy level	0.12	0.26	3.21	0.01
Knowledge- and skill-based approach to health (OHLA)	0.17	0.43	3.38	0.01
Willingness and responsibility for occupational health (OHLB)	0.06	0.55	1.36	0.17
Participation possibilities in health (PART)	0.19	0.32	3.34	0.01
OHLA*PART^a^	−0.11	0.46	−2.22	0.03
OHLB*PART^b^	0.10	0.53	2.01	0.04

^a^OHLA*PART, Interaction between the factor knowledge- and skill-based approach to health and participation possibilities in health. ^b^OHLB*PART, Interaction between the factor willingness and responsibility for occupational health and participation possibilities in health.

**Figure 4:**
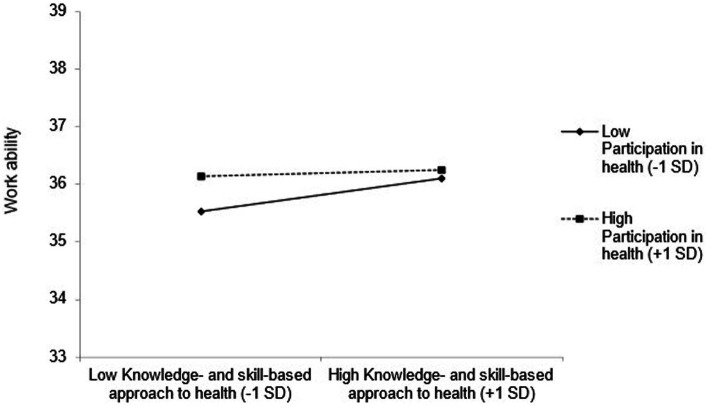
The moderating effect of the knowledge- and skill-based approach to health and participation possibilities in health on work ability.

**Figure 5:**
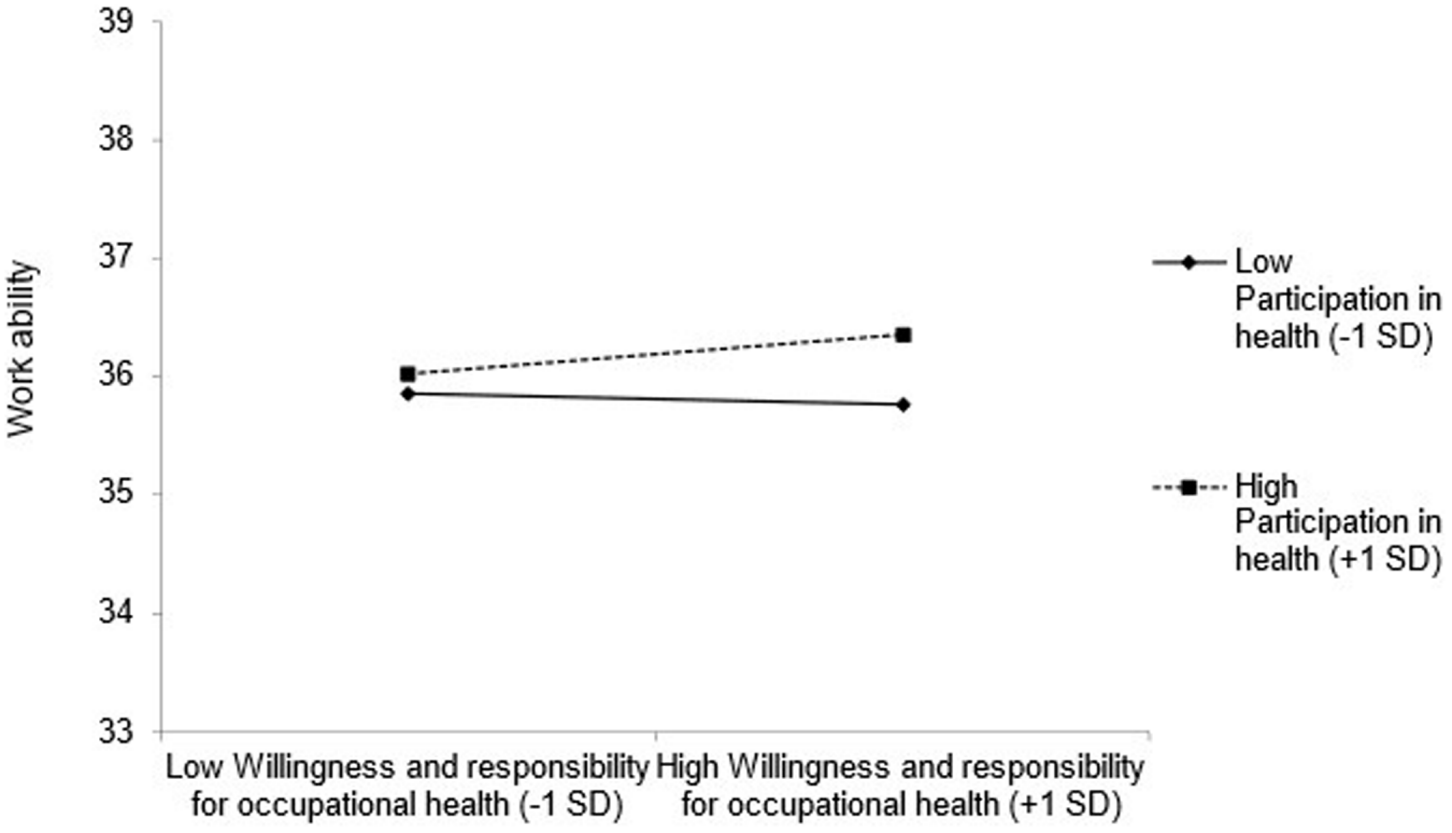
The moderating effect of willingness and responsibility for occupational health and participation possibilities in health on work ability.

The slope for employees who perceived fewer participation possibilities (*B* = 2.30, *p* < 0.01) was steeper than for those who perceived more participation possibilities (*B* = 0.60, *p* = 0.25) regarding the influence of the factor OHLA on WAI (see Figure 4). For employees with lower OHLB, the relationship between participation possibilities and work ability was not statistically significant (see Figure 5). However, employees with higher OHLB, those with more perceived participation possibilities (*B* = 1.64, *p* = 0.02), also reported higher work ability compared to those who perceived fewer participation possibilities (*B* = −0.15, *p* = 0.82).

Finally, the same latent moderation analysis was conducted for OHL factors as independent variables, values of health in companies as moderator, and WAI as a dependent variable. Fit indices were in an acceptable range: χ^2^(743) = 1472.2, *p* < 0.001, CFI = 0.91, TLI = 0.89, RMSEA = 0.04, SRMR = 0.06. While a significant main effect of values of health in companies was observed (*B* = 0.20, *SE* = 0.40, *p* < 0.01), no significant moderation effects were found. Therefore, values of health in companies did not moderate the relationship between OHL factors and WAI. An overview of all hypotheses, investigated scales, and statistically significant effects partly supporting the hypotheses can be found in Table 6.

**Table 6:** Hypotheses and effects of investigated scales.

Hypothesis	Investigated scales	Significant effect found?
1. The individual and interpersonal factors (OHL and HoL), as well as the organizational factors (participation possibilities in health and values of health in companies), are positively associated with work ability when controlling for age, gender, education, and hierarchy level.	OHLA^a^	Yes
	OHLB^b^	No
	HoL^c^	Yes
	PART^d^	Yes
	VAL^e^	Yes
2. On an interpersonal level, health-oriented leadership moderates the positive relationship between OHL and work ability.	OHLA*HoL	Yes
	OHLB*HoL	No
3. On an organizational level, participation possibilities in health and values of health in companies moderate the positive relationship between OHL and work ability.	OHLA*PART	Yes
	OHLB*PART	Yes
	OHLA*VAL	No
	OHLB*VAL	No

^a^OHLA, Factor knowledge- and skill-based approach to health; ^b^OHLB, Willingness and responsibility for occupational health; ^c^HoL health-oriented leadership; ^d^PART, Participation possibilities in health at work; and ^e^VAL, Values of health in companies.

## 4 Discussion

This study aimed to examine the relationship between OHL (on an individual level) and work ability including the moderating effects of HoL (interpersonal level) and participation possibilities in health and values of health (organizational level). OHL comprised two factors: a knowledge- and skill-based approach to health and a willingness and responsibility for occupational health. Except for the factor willingness and responsibility for occupational health, all factors, namely knowledge- and skill-based approach to health, HoL, participation possibilities in health, and values of health in companies were significantly positively related to the WAI. Thus, hypothesis 1 was almost fully supported. HoL moderated the positive relationship between the WAI and the first OHL factor knowledge- and skill-based approach to health but not the second OHL factor willingness and responsibility for occupational health, partly confirming hypothesis 2. Furthermore, participation possibilities in health moderated the relationship between both OHL factors and the WAI while values of health in companies did not. Therefore, hypothesis 3 was partly supported.

### 4.1 Work ability and its relationships

We found significant relationships between work ability and the individual factors. The positive relationship between general health literacy and work ability was also assessed in a study by Gernert et al. (29) which is in line with our findings with a domain-specific scale of OHL. While the authors found no direct effect of the proactive approach to health on work ability (29), our OHL factor willingness and responsibility for occupational health showed also no significant relationship. It seems that a motivational component or proactive approach to health indirectly affects work ability via dealing with health information or self-regulatory competencies (34). More active employees in health seem to better anticipate health-promoting situations and deal with health information. With applying health information, unhealthy behaviors could be diminished, which has positive influences on health status, well-being, or in the long term work ability (33, 80).

Despite the individual factors, it is important to consider other factors on an interpersonal level. Like the communication and cooperation in companies (34), a HoL approach showed also a significant relationship with work ability on an interpersonal level. A perceived HoL can be seen as a kind of social support in health issues, which is also associated with an improvement in work ability (81).

Moreover, cooperative and social interactions should be taken into account while creating health promotion (34), which is in line with the positive relationship between organizational factors like increased participation possibilities in health and work ability. When employees feel involved and have a say in changing work situations (82), they can better balance the dynamic between job demands and their individual health or competencies (14). Furthermore, when considering work environmental factors in healthy organizations (6), improvements in occupational health management can have a greater impact on improving work ability (19). We defined the values of health in companies as better perceived conditions at work and the perceived financial capacities of a company. These conditions were positively related to individual work ability in our study, which strengthens the integration of a holistic approach in healthy organizations (10): Changing the workplace into a healthy place would lead to better perceptions of conditions and better work ability.

### 4.2 OHL and moderation on an interpersonal level on work ability

Moderating effects on the relationship between OHL and WAI were investigated on an interpersonal level. A moderating effect of HoL was only observed for the knowledge- and skill-based approach to health and not for the willingness and responsibility for occupational health of the OHL scale. This indicates that when competencies regarding occupational health are high, HoL is less decisive, but when competencies are low, people profit more from HoL. HoL emphasizes that leaders affect employee health in multiple ways, directly through their communication and behavior and indirectly by influencing tasks and working conditions (43, 83). Therefore, on an interpersonal level, HoL plays a crucial role in the acquisition of knowledge and skills in occupational health and supports employees with low OHL. An interplay of these factors elucidates that both the employee and the employer are crucial for supporting work ability. This result has implications for occupational health promotional efforts to not only place the responsibility for improving work ability on the individual but also to create a health-oriented environment and support the individuals in participating through positive leadership (43, 84, 85). Thus, the interaction can considerably improve work ability if the managers exemplify healthy working, live up to their role model function, and keep the employees’ health in mind. These results contribute specific empirical evidence that corroborates or supports the theoretical assumptions.

### 4.3 OHL and moderation on an organizational level on work ability

Additionally, we put forward that participation possibilities in health at work and values of health in companies moderate the positive relationship between OHL factors and WAI on an organizational level. Only the participation possibilities in health were observed to have a statistically significant interaction with both OHL factors. Values of health at work did not have a statistically significant interaction. We assumed, that when having the possibility to invest in health promotion and adjusting for negative health-related environmental factors as a company, the employees could navigate and behave in a healthy way in the organization which could lead to better work ability (82). These results indicate that a participatory health-oriented work environment was more decisive for work ability (34) than only the organizational framework for the health of the companies (82) in our sample.

Although organizational conditions for changing health at work and a financial budget for health promotion exist, it is not yet certain that employees will participate in the measures offered and those lead to better health for employees. A person-oriented approach to health promotion seems to make it possible to relate the existing environmental factors to the employee and to find individual solutions together with the employer and employee to maintain their ability to work. If the needs and resources of employees are recognized, health programs can be adapted to specific target groups so that the organizational conditions change and affect personal competencies and work ability (86).

In the case of low knowledge and skills concerning occupational health, employees differed in their reported work ability depending on perceived participation possibilities. However, in the case of high knowledge and skills, the perception of high or low participation possibilities did not affect the employees’ work ability. As a possible explanation, participation possibilities in health at work are associated with the knowledge and understanding of the interrelationships of health and sources of information (87). The degree of input from leaders or the organization and participation in decision-making might be the moderating roles for higher work ability. These results might imply integrating a participatory design theory (88) to improve health literacy models and interventions to have an even higher effect on work ability in the long term. In turn, greater participation and decision-making or involvement in health processes can relieve the burden on employers (especially in small- and medium-sized enterprises) and can strengthen the health literacy of employees (89).

### 4.4 Limitations

We captured employers and employees working in small and medium-sized enterprises from a pool of interested people in Germany with computer-assisted telephone interviews. Participants may report more positively on a telephone rather than on a paper-pencil or computer-based questionnaire, resulting in higher values (e.g., for OHL). Due to the respondents being anonymous, we were not able to account for certain organizational-level variables or potential hierarchal structures in the tested models. Due to the focus on small and medium-sized enterprises an adjustment by size was not made and has to be taken into account while interpreting and generalizing the results. Furthermore, within this cross-sectional study, no causal effects can be driven. We showed different significant associations of individual, interpersonal, and organizational levels with WAI, but further longitudinal analyses or randomized controlled trials should examine changes and direction of effects over time.

Second, parts of the measurements were self-constructed within this study. To measure participation possibilities in health at work or values of health in companies, to our knowledge, the questionnaires available in German are rare or do not capture the constructs and our conceptual definitions. The factor structure, fit indices, and internal consistencies for OHL (25) and participation possibilities in health are adequate within the study results. For HoL, we used three adapted items from the original scale due to their better contextuality and economy within the study. Ideally, the complete scale for HoL could have been used. Nevertheless, internal consistency was high (*α* = 0.90), indicating an appropriate selection of items. The self-constructed scale for values of health in companies had a low internal consistency and the scale needs further research adjustments. Therefore, the non-significant effect might occur due to the measurement and should be carefully interpreted.

Third, multicollinearity between work-related health factors was a limitation. The OHL scale showed two highly correlated factors, as both factors are relevant for the whole model while each maintaining its unique impact. We solved some of these problems while analyzing the individual and organizational factors using separate latent regression models.

### 4.5 Implications for research and practice

Future research could focus on the survey gap on participation possibilities in health and the importance of health values in companies. Models and survey instruments would facilitate measuring and examining whereby these self-constructed questions can be understood as a first impulse to orient research on health at work in a diversity-sensitive and participatory way. Furthermore, conditions for employees can be strengthened at various levels. For healthy organizations, the importance of mission statements related to health should be highlighted. In unhealthy and unsafe workplaces, accidents or work-related illnesses can occur more frequently, leading to absenteeism or decreased productivity. Specific measures at the organizational level are particularly worthwhile for individuals with low competencies. Suppose health in the organization is lived through participation and a positive, health-promoting environment. In that case, it is easier for people with fewer resources to adopt healthy behavior and remain able to work in the long term. On the one hand, it is important to think of OHL together with an interpersonal factor such as HoL and implement it in health interventions at work. The findings have implications for interventions, suggesting that all levels of an organization should be addressed to achieve comprehensive change regarding health. In addition, participation possibilities in health at work are important for tailoring interventions or strategies to specific conditions and optimizing their effectiveness in the work context. On the other hand, framework conditions on the organizational level are indispensable for individuals to be able to commit themselves to health at work and to participate in a healthy work environment. In making specific positive changes to the work environment, points of entry could be found in inquiring about current needs and idea management, both working toward health improvement in companies. This might lead to greater awareness of participation opportunities and better work ability, less absenteeism, and/or higher productivity in the long term.

## 5 Conclusion

Employees with low levels of OHL benefit in terms of their ability to work from HoL and from possibilities to participate in health. If employees can participate, they are motivated to change their own workplace or that of their colleagues into a healthy work environment. These findings emphasize the importance of creating a supportive work environment on an individual, interpersonal, and organizational level. Employers, stakeholders, and policymakers should be aware of providing employees with adequate OHL, HoL, and participation opportunities to maintain and improve work ability and change the work environment into a healthy place.

## Data availability statement

The datasets presented in this article are not readily available because of the ongoing research project. Requests to access the datasets should be directed to gorden.sudeck@uni-tuebingen.de.

## Ethics statement

The studies involving humans were approved by Ethics Commission of the Charité—Universitätsmedizin Berlin (reference numbers: EA2/098/19 and EA4/133/20). The studies were conducted in accordance with the local legislation and institutional requirements. The participants provided their written informed consent to participate in this study.

## Author contributions

JF and MR conceived and designed the manuscript idea and wrote the manuscript draft. JF analyzed the data. Y-SF assisted with the data analysis. JF, MR, Y-SF, and GS provided substantial input and critical revision of the manuscript. All authors contributed to the article and approved the submitted version.
