# Supplementary material for: Occupational health literacy and work ability: a moderation analysis including interpersonal and organizational factors in healthy organizations
Source: Front Public Health. 2024 Feb 7;12:1243138. doi: 10.3389/fpubh.2024.1243138 (PMC10879437; doi:10.3389/fpubh.2024.1243138)
Supplement: Supplementary file 1 [file Data_Sheet_1.PDF]

Supplementary Material

Occupational health literacy and work ability: A moderation analysis including interpersonal and organizational factors in healthy organizations

Julian Friedrich[1*†], Maylin Rupp[2†], You-Shan Feng[3], Gorden Sudeck[1]

*** Correspondence:** Julian Friedrich: julian.friedrich@tum.de

Table S1. Means (M), standard deviations (SD), Cronbach's alphas (α) of the scales and standardized factor covariances.

Factors	*M*	*SD*	α	1	2	3	4	5
1. Work ability	36.05	4.82						
2. Knowledge- and skill-based approach to health	33.50	9.90	.88	.35**				
3. Willingness and responsibility for occupational health	38.11	10.07	.75	.13*	.34**			
4. Health-oriented leadership	3.44	1.12	.90	.28**	.44**	.33**		
5. Participation in health	3.71	1.00	.82	.30**	.53**	.43**	.65**	
6. Values of health in companies	3.69	0.89	.52	.27**	.50**	.31**	.53**	.61**

Note. * indicates $p < .05$. ** indicates $p < .01$.
